# Supplementary material for: End Sequence Analysis Toolkit (ESAT) expands the extractable information from single-cell RNA-seq data
Source: Genome Res. 2016 Oct;26(10):1397–410. doi: 10.1101/gr.207902.116 (PMC5052061; doi:10.1101/gr.207902.116)
Supplement: Supplemental Material [file supp_gr.207902.116_Supp_Materials.tar.gz › ESAT-esat_v0.1/doc/html/classbroad_1_1pda_1_1seq_1_1rap_1_1_collect_annotation_enrichments.html]

ESAT: broad.pda.seq.rap.CollectAnnotationEnrichments Class Reference


ESAT


- Main Page
- Related Pages
- Packages
- Classes
- Files

- Class List
- Class Index
- Class Hierarchy
- Class Members

All Classes Namespaces Files Functions Variables Enumerator Pages

- broad
- pda
- seq
- rap
- CollectAnnotationEnrichments

Classes |
Static Public Member Functions |
Public Attributes |
Protected Member Functions |
List of all members

broad.pda.seq.rap.CollectAnnotationEnrichments Class Reference

Inheritance diagram for broad.pda.seq.rap.CollectAnnotationEnrichments:

[legend]

Collaboration diagram for broad.pda.seq.rap.CollectAnnotationEnrichments:

[legend]

|  |  |
| --- | --- |
| Classes | |
| class | **OverlapEnrichmentScore** |
|  | |

|  |  |
| --- | --- |
| Static Public Member Functions | |
| static void | main (final String[] args) |
|  | |

|  |  |
| --- | --- |
| Public Attributes | |
| String | USAGE |
|  | |
| File | INPUT |
|  | |
| List< File > | ANNOTATIONS |
|  | |
| String | ANALYSIS\_NAME |
|  | |
| File | OUTPUT |
|  | |
| Integer | PERMUTATIONS = 100 |
|  | |
| boolean | KEEP\_INTERMEDIATES = false |
|  | |
| String | QUEUE = null |
|  | |
| boolean | PERMUTE = true |
|  | |
| String | ANNOTATION\_BASE = "" |
|  | |
| List< String > | DISCRETE\_EXTENSION = new ArrayList<String>() |
|  | |
| List< String > | BAM\_EXTENSION = new ArrayList<String>() |
|  | |
| List< String > | CONTINUOUS\_EXTENSION = new ArrayList<String>() |
|  | |
| boolean | PAIRED\_END = true |
|  | |
| Public Attributes inherited from broad.pda.seq.rap.GenomeCommandLineProgram | |
| String | SIZES = "/seq/lincRNA/data/mm9/sizes" |
|  | |
| String | MASK\_FILE = "/seq/mguttman/ChIPData/MaskFiles/MM9Segments/all.mask.mouse.n36.d2.bin" |
|  | |
| double | PCT\_MASKED\_ALLOWED = 50.0 |
|  | |
| String | REGION = null |
|  | |
| String | GENE = null |
|  | |
| Integer | MAX\_FRAGMENT\_LENGTH = 10000 |
|  | |
| Integer | MIN\_MAPPING\_QUALITY = 30 |
|  | |
| String | COORD\_SPACE = "genomic" |
|  | |
| String | ANNOTATION = "/seq/lincRNA/Shari/Annotations/RefSeq\_LincV3\_ChromatinWithNames\_nonrandom\_collapsed\_overlappers.bed" |
|  | |

|  |  |
| --- | --- |
| Protected Member Functions | |
| String[] | customCommandLineValidation () |
|  | |
| int | doWork () |
|  | |
| void | shufflePeaks (AnnotationList< Annotation > regions, AnnotationList< Annotation > peaks, String output) throws IOException |
|  | |
| void | submitJobs (SortedMap< String, File > annotationMap) throws IOException, InterruptedException, DrmaaException |
|  | |
| void | calculateDiscreteAnnotationEnrichments (AnnotationList< Annotation > regions, AnnotationList< Annotation > peaks, SortedMap< String, File > discreteAnnotations) throws IOException |
|  | |
| void | calculateBamEnrichments (AnnotationList< Annotation > regions, AnnotationList< Annotation > peaks, SortedMap< String, File > bamMap) throws IOException |
|  | |
| void | processOneAnnotationSet (String name, AnnotationList< Annotation > regions, AnnotationList< Annotation > peaks, AnnotationCollection<?extends Annotation > annotations) throws IOException |
|  | |
| void | collectResults (SortedMap< String, File > discreteAnnotations) throws IOException |
|  | |
| void | cleanup (SortedMap< String, File > discreteAnnotations) |
|  | |
| List< CountScore > | getPeakScores (AnnotationList< Annotation > regions, AnnotationList<?extends Annotation > peaks, AnnotationCollection<?extends Annotation > annotations) |
|  | |
| Protected Member Functions inherited from broad.pda.seq.rap.GenomeCommandLineProgram | |
| String[] | customCommandLineValidation () |
|  | |
| void | loadCoordinateSpace () throws IOException |
|  | |
| CoordinateSpace | getCoordinateSpace () |
|  | |

|  |  |
| --- | --- |
| Additional Inherited Members | |
| Public Member Functions inherited from broad.pda.seq.rap.GenomeCommandLineProgram | |
| List< Annotation > | getRegions () |
|  | |
| Map< String, Annotation > | getRegionsMap () |
|  | |
| AnnotationList< Annotation > | getRegionSet () |
|  | |
| Annotation | getGenes () |
|  | |
| Boolean | isGenomicSpace () |
|  | |
| AlignmentModel | loadAlignmentModel (File bamFile) |
|  | |
| AlignmentModel | loadAlignmentModel (File bamFile, boolean pairedEnd) |
|  | |
| Protected Attributes inherited from broad.pda.seq.rap.GenomeCommandLineProgram | |
| CoordinateSpace | coordinateSpace |
|  | |

## Member Function Documentation

|  |  |  |  |
| --- | --- | --- | --- |
| void broad.pda.seq.rap.CollectAnnotationEnrichments.calculateBamEnrichments | ( | AnnotationList< Annotation > | *regions*, |
|  |  | AnnotationList< Annotation > | *peaks*, |
|  |  | SortedMap< String, File > | *bamMap* |
|  | ) |  | throws IOException |

protected

Here is the call graph for this function:

Here is the caller graph for this function:

|  |  |  |  |
| --- | --- | --- | --- |
| void broad.pda.seq.rap.CollectAnnotationEnrichments.calculateDiscreteAnnotationEnrichments | ( | AnnotationList< Annotation > | *regions*, |
|  |  | AnnotationList< Annotation > | *peaks*, |
|  |  | SortedMap< String, File > | *discreteAnnotations* |
|  | ) |  | throws IOException |

protected

Here is the call graph for this function:

Here is the caller graph for this function:

|  |  |  |  |  |  |
| --- | --- | --- | --- | --- | --- |
| void broad.pda.seq.rap.CollectAnnotationEnrichments.cleanup | ( | SortedMap< String, File > | *discreteAnnotations* | ) |  |

protected

Here is the caller graph for this function:

|  |  |  |  |  |  |
| --- | --- | --- | --- | --- | --- |
| void broad.pda.seq.rap.CollectAnnotationEnrichments.collectResults | ( | SortedMap< String, File > | *discreteAnnotations* | ) | throws IOException |

protected

Here is the caller graph for this function:

|  |  |  |  |  |
| --- | --- | --- | --- | --- |
| String [] broad.pda.seq.rap.CollectAnnotationEnrichments.customCommandLineValidation | ( |  | ) |  |

protected

|  |  |  |  |  |
| --- | --- | --- | --- | --- |
| int broad.pda.seq.rap.CollectAnnotationEnrichments.doWork | ( |  | ) |  |

protected

Here is the call graph for this function:

|  |  |  |  |
| --- | --- | --- | --- |
| List<CountScore> broad.pda.seq.rap.CollectAnnotationEnrichments.getPeakScores | ( | AnnotationList< Annotation > | *regions*, |
|  |  | AnnotationList<?extends Annotation > | *peaks*, |
|  |  | AnnotationCollection<?extends Annotation > | *annotations* |
|  | ) |  |  |

protected

Here is the caller graph for this function:

|  |  |  |  |  |  |
| --- | --- | --- | --- | --- | --- |
| static void broad.pda.seq.rap.CollectAnnotationEnrichments.main | ( | final String[] | *args* | ) |  |

static

Stock main method.

Parameters
:   |  |  |
    | --- | --- |
    | args | main arguments |

|  |  |  |  |
| --- | --- | --- | --- |
| void broad.pda.seq.rap.CollectAnnotationEnrichments.processOneAnnotationSet | ( | String | *name*, |
|  |  | AnnotationList< Annotation > | *regions*, |
|  |  | AnnotationList< Annotation > | *peaks*, |
|  |  | AnnotationCollection<?extends Annotation > | *annotations* |
|  | ) |  | throws IOException |

protected

Here is the call graph for this function:

Here is the caller graph for this function:

|  |  |  |  |
| --- | --- | --- | --- |
| void broad.pda.seq.rap.CollectAnnotationEnrichments.shufflePeaks | ( | AnnotationList< Annotation > | *regions*, |
|  |  | AnnotationList< Annotation > | *peaks*, |
|  |  | String | *output* |
|  | ) |  | throws IOException |

protected

Here is the call graph for this function:

Here is the caller graph for this function:

|  |  |  |  |  |  |
| --- | --- | --- | --- | --- | --- |
| void broad.pda.seq.rap.CollectAnnotationEnrichments.submitJobs | ( | SortedMap< String, File > | *annotationMap* | ) | throws IOException, InterruptedException, DrmaaException |

protected

Here is the caller graph for this function:

## Member Data Documentation

String broad.pda.seq.rap.CollectAnnotationEnrichments.ANALYSIS\_NAME

String broad.pda.seq.rap.CollectAnnotationEnrichments.ANNOTATION\_BASE = ""

List<File> broad.pda.seq.rap.CollectAnnotationEnrichments.ANNOTATIONS

List<String> broad.pda.seq.rap.CollectAnnotationEnrichments.BAM\_EXTENSION = new ArrayList<String>()

List<String> broad.pda.seq.rap.CollectAnnotationEnrichments.CONTINUOUS\_EXTENSION = new ArrayList<String>()

List<String> broad.pda.seq.rap.CollectAnnotationEnrichments.DISCRETE\_EXTENSION = new ArrayList<String>()

File broad.pda.seq.rap.CollectAnnotationEnrichments.INPUT

boolean broad.pda.seq.rap.CollectAnnotationEnrichments.KEEP\_INTERMEDIATES = false

File broad.pda.seq.rap.CollectAnnotationEnrichments.OUTPUT

boolean broad.pda.seq.rap.CollectAnnotationEnrichments.PAIRED\_END = true

Integer broad.pda.seq.rap.CollectAnnotationEnrichments.PERMUTATIONS = 100

boolean broad.pda.seq.rap.CollectAnnotationEnrichments.PERMUTE = true

String broad.pda.seq.rap.CollectAnnotationEnrichments.QUEUE = null

String broad.pda.seq.rap.CollectAnnotationEnrichments.USAGE

**Initial value:**

= "Calculate overlap/count enrichments for regions in the query BED file. " +

"Significance is assessed by permuting the query BED file. " +

"Regions in the BED file can be scored against other BED files or against BAM files."

---

The documentation for this class was generated from the following file:

- java/broad/pda/seq/rap/CollectAnnotationEnrichments.java


---

Generated on Fri Aug 15 2014 15:11:03 for ESAT by  

 1.8.7
